# Homeostatic Influence of Fig4 Outside of the Fab1‐Vac14‐Fig4 Complex in *Saccharomyces cerevisiae*

**DOI:** 10.1111/mmi.70018

**Published:** 2025-07-31

**Authors:** Hannah E. Reeves, Anna King, Imran Khan, Asha Thomas, Corey Chung, Anirudan Sivaprakash, Harrison A. Hall, Cole McGuire, Victoria Cruz, Alim Habib, Lauren Dotson, Sophia R. Lee, Caroline L. Darbro, Bethany S. Strunk

**Affiliations:** ^1^ Department of Biology Trinity University San Antonio Texas USA; ^2^ Life Sciences Institute University of Michigan Ann Arbor Michigan USA

**Keywords:** phosphatidylinositols, phosphoric monoester hydrolases, *Saccharomyces cerevisiae*, sirolimus

## Abstract

The lipid phosphatase Fig4 is conserved in all eukaryotes and is associated with human neurological diseases for which there are currently no specific therapies. Fig4 functions in both the production and turnover of its lipid substrate, PI3,5P2, through participation in the Fab1‐Vac14‐Fig4 complex with its opposing kinase Fab1. The molecular mechanisms through which Fig4 influences PI3,5P2 production are not fully understood but are believed to require Fig4 binding to the scaffold protein Vac14. We unexpectedly found that multiple Fig4 disease‐related mutants that are impaired in binding to the Fab1‐Vac14‐Fig4 complex dominantly confer tolerance to rapamycin, an inhibitor of the Target of Rapamycin Complex 1 (TORC1), when expressed in *Saccharomyces cerevisiae* . Fig4‐dependent rapamycin tolerance is conferred under moderate heat stress, independent of Vac14 and Fig4 catalytic activity. Conversely, expression of catalytically dead Fig4 that binds stably to the Fab1‐Vac14‐Fig4 complex enhances rapamycin sensitivity. We propose that Fig4 disease‐related mutants alter TORC1 signaling through gain of function under these conditions through an abnormal or sustained interaction with an unknown factor, perhaps by altering PI3,5P2 production. Investigation of the mechanisms whereby Fig4 mutants alter rapamycin tolerance may provide new insights into Fig4 molecular functions with potential relevance for Fig4‐related diseases.

## Graphical abstract

Disease‐related Fig4 mutants displaying impaired association with the Fab1‐Vac14‐Fig4 complex confer rapamycin tolerance in *Saccharomyces cerevisiae* independent of Vac14 and Fig4 catalytic function. Fig4 may thus modulate homeostasis through uncharacterized molecular interactions that lead to altered function when not bound to Vac14.

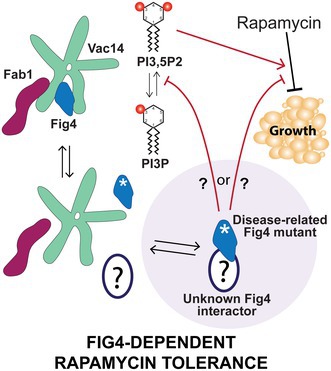

## 1 Introduction

Phosphoinositide (PI) lipid phosphatases modulate cellular signaling cascades through localized dephosphorylation of specific PI lipids on subcellular membrane domains. The PI phosphatase Fig4 (alpha factor–induced gene 4) (Erdman et al. 1998) is conserved in all eukaryotes and is important both for development and long‐term homeostasis in mammals (Chow et al. 2007; Lenk et al. 2016; Mironova et al. 2016, 2018). Biallelic mutations in Fig4 are associated with multiple human genetic disorders involving demyelination in the peripheral or central nervous system (Chow et al. 2007, 2009; Campbell and Mahad 2011; Baulac et al. 2014; Hu et al. 2018; Lenk et al. 2019). Fig4 influences cellular homeostasis through regulation of its lipid substrate, phosphatidylinositol 3,5‐bisphosphate (PI3,5P2), a low abundance PI found primarily on late endosomal and lysosomal membranes in mammalian cells and on the vacuole in yeast (Rivero‐Ríos and Weisman 2022; Barlow‐Busch et al. 2023). Local elevation or depletion of PI3,5P2 modulates diverse cellular pathways via specific binding interactions between PI3,5P2 and a variety of protein effectors. Pathways regulated by PI3,5P2 include control of actin dynamics, membrane homeostasis, and signaling in response to stress (Rivero‐Ríos and Weisman 2022).

**FIGURE 1:**
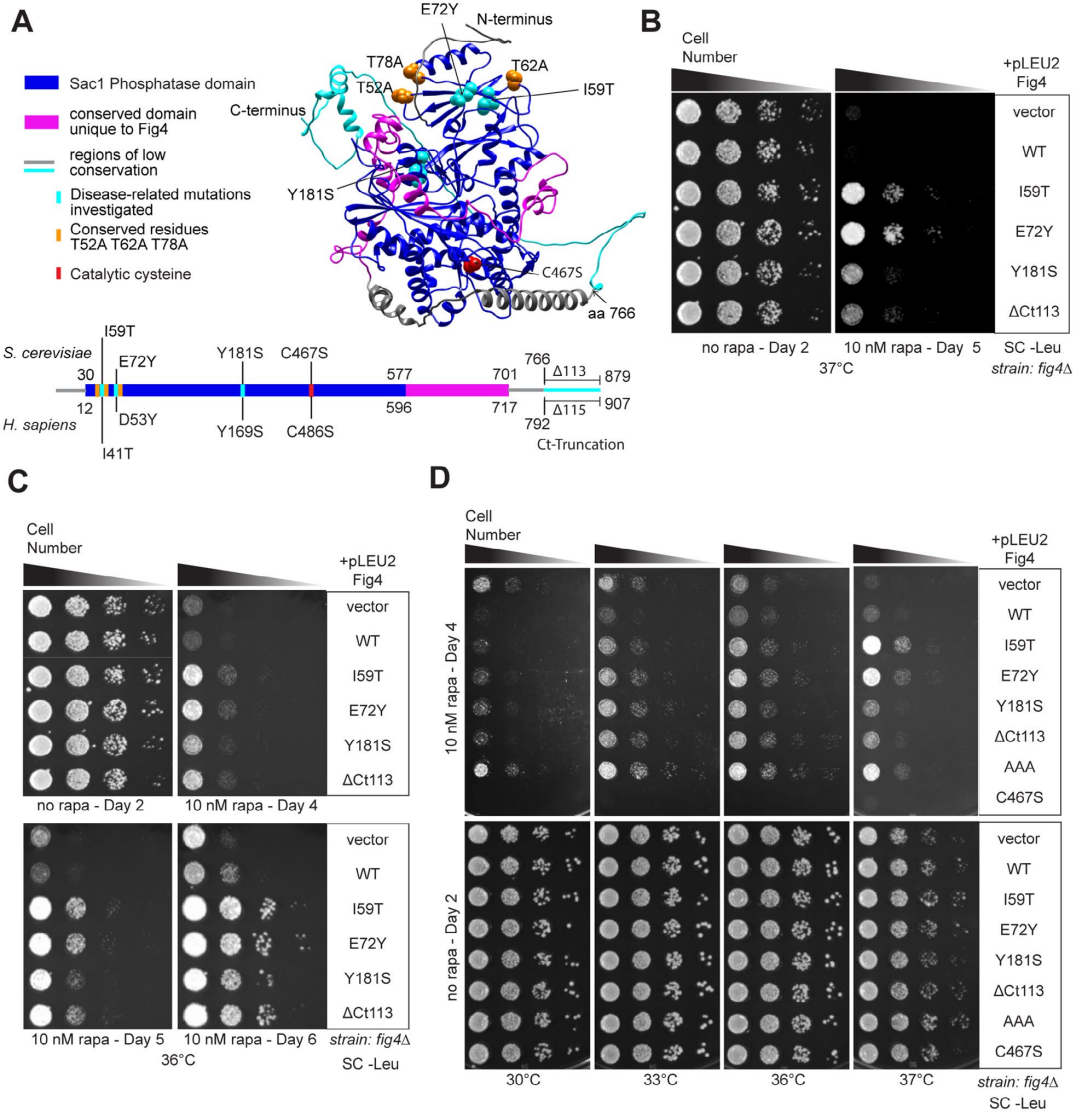
Disease‐related mutants of Fig4 confer tolerance to rapamycin at elevated temperatures. (A) Linear schematic of Fig4 primary sequence and Alphafold3 predicted structure of Fig4‐WT (Abramson et al. 2024). Conservation between *Saccharomyces cerevisiae* (top) and *H. sapiens* (bottom) indicated. Residues mutated in this study are colored as follows: Disease related mutations I59T (CMT4J), E72Y (ALS), Y181S and truncated amino acids C‐terminal to amino acid 766 in ΔCt113 (leukodystrophy) in cyan, Fig4‐AAA mutations (T52A, T62A, and T78A) in orange, active site Fig4‐C467S in red. (B) Multiple disease‐related mutants confer rapamycin tolerance at 37°C compared to wild‐type or no Fig4. (C) Cells expressing Fig4 WT or no Fig4 continue to grow slowly relative to disease mutants through Day 6 at 36°C. (D) Rapamycin tolerance is restricted to growth temperatures above 33°C. The Vac14 binding‐impaired mutant Fig4‐AAA behaves similarly to disease‐related Fig4 mutants with regard to rapamycin tolerance. Catalytically dead Fig4‐C467S confers rapamycin sensitivity at all temperatures. For growth assays in (B–D) a *fig4Δ* strain transformed with the plasmids indicated was spotted on agar plates grown under the conditions specified. Fig4 variants in this figure: Wild‐type (WT), CMT4J (I59T), ALS (E72Y), and leukodystrophy (Y181S and ΔCt113), T52A‐T62A‐T78A (AAA), catalytically‐dead (C467S), or no Fig4 (vector). All genes expressed from plasmids with native 5′ and 3′ regulatory regions.

Fig4 plays two opposing roles in the regulation of PI3,5P2. Fig4 phosphatase activity reduces levels of PI3,5P2 by converting it to PI3P through dephosphorylation of the 5‐position of the inositol ring (Rudge et al. 2004; Lees et al. 2020). Fig4 also promotes the dynamic elevation of PI3,5P2 through physical association with its opposing PI 5‐kinase, Fab1, the sole enzyme known to catalyze PI3,5P2 production in eukaryotic cells (Gary et al. 1998; Sbrissa et al. 2007, 2008; Botelho et al. 2008; Jin et al. 2008) (Figure 1A). The latter function explains why Fig4 null mutants are counterintuitively characterized by PI3,5P2 insufficiency rather than elevation of this lipid substrate (Duex, Nau, et al. 2006; Chow et al. 2007). Fig4 loss of function mutations are accordingly associated with enlarged vacuoles, a hallmark of PI3,5P2 insufficiency in eukaryotes from yeast to mammals (Rudge et al. 2004; Duex, Tang, and Weisman 2006; Chow et al. 2007; Bharadwaj et al. 2016). The molecular mechanisms by which Fig4 controls PI3,5P2 production and turnover are not fully understood, but available data suggest that both processes occur exclusively through the stable and direct binding of Fig4 to the scaffold protein Vac14 (Lees et al. 2020; Rivero‐Ríos and Weisman 2022).

Fig4 is localized to the limiting membrane of endosomal compartments through binding to Vac14 (Rudge et al. 2004; Jin et al. 2008; Bharadwaj et al. 2016). Moreover, Vac14 binding to Fig4 is required for both of these proteins to stably associate with Fab1 in the assembly of the Fab1‐Vac14‐Fig4 complex (Sbrissa et al. 2007, 2008; Botelho et al. 2008; Jin et al. 2008; Lees et al. 2020). Fig4 null phenotypes are often milder versions of those caused by the loss of Vac14 (Duex, Nau, et al. 2006; Chow et al. 2007; Lines et al. 2017; Lenk et al. 2019; Cao et al. 2023). Accordingly, phenotypes associated with Fig4 mutations are attributed to dysregulation of PI3,5P2 through impaired function of the Fab1‐Vac14‐Fig4 complex. In yeast, Fig4 is not required for basal production of PI3,5P2, but stable binding of Fig4 to the Fab1‐Vac14‐Fig4 complex is required for elevation of PI3,5P2 in response to hyperosmotic shock (Chow et al. 2007; Strunk et al. 2020). Fig4 can support PI3,5P2 production independent of its catalytic function; however, catalytically dead Fig4 mutants do not support the full induction of PI3,5P2 following hyperosmotic shock in *Saccharomyces cerevisiae* (Duex, Tang, and Weisman 2006; Strunk et al. 2020). Predicted protein phosphatase activity of Fig4 may also contribute to the regulation of PI3,5P2 production (Lees et al. 2020).

Proteins regulated through direct binding to PI3,5P2 include the master regulator of growth and metabolism, target of rapamycin kinase complex 1 (TORC1) (McCartney et al. 2014). When TORC1 is active, it promotes growth and cell proliferation through phosphorylation of multiple substrates that serve to activate translation, upregulate the protein synthetic machinery, and counteract catabolic processes (Foltman and Sanchez‐Diaz 2023; Khalil et al. 2023). Conversely, when TORC1 is inactive, catabolic processes are activated and growth is slowed or arrested in association with reduced protein synthesis, inhibition of cell cycle progression, and the initiation of stress‐related transcription programs (Loewith and Hall 2011; Saxton and Sabatini 2017; Ben‐Sahra and Manning 2017; Kim and Guan 2019). Cycles of TORC1 activation and inhibition are normal aspects of the cell cycle and developmental programs (Guerra et al. 2022; Foltman and Sanchez‐Diaz 2023). Sustained TORC1 inhibition is naturally triggered by starvation and stress conditions including hypoxia and osmotic stress (Nakazawa et al. 2016; Weisman 2016; Schito and Rey 2018; Lee et al. 2019). TORC1 can be inhibited pharmaceutically by the natural macrolide rapamycin (Saxton and Sabatini 2017; Panwar et al. 2023).

The conservation of the PI3,5P2 regulatory machinery between *S. cerevisiae* and humans has allowed the use of this microbial model system to investigate molecular mechanisms of PI3,5P2 production and turnover with relevance to human biology. In several cases, mutations in Fig4 associated with different human diseases are in residues conserved from yeast to humans. Yeast Fig4‐I59T corresponds to human I41T, which is associated with Charcot–Marie–Tooth disease type 4J (CMT4J), amyotrophic lateral sclerosis (ALS), and primary lateral sclerosis (PLS) (Chow et al. 2007; Osmanovic et al. 2017; de Boer et al. 2023; Mendes Ferreira et al. 2024). Fig4‐E72Y corresponds to human D53Y, which is associated with ALS (Chow et al. 2009; Stump et al. 2023). Fig4‐Y181S corresponds to human Y169S, which is associated with leukodystrophy (Lenk et al. 2019) (Figure 1A). The conservation of these sites across evolutionary taxa supports the potential for conservation of Fig4 function as well.

Here we report evidence that, in *S. cerevisiae* , Fig4 can contribute to cellular homeostasis outside of its known modes of interaction with the Fab1‐Vac14‐Fig4 complex. This comes through the unexpected finding that disease‐related mutants of Fig4 confer tolerance to rapamycin when cells are grown under mild heat stress. This phenotype is conferred by Fig4 mutants that display impaired binding to the Fab1‐Vac14‐Fig4 complex and is independent of Fig4 catalytic function. Conversely, expression of catalytically dead Fig4 that stably binds Vac14 results in rapamycin sensitivity. Importantly, when Vac14 is absent, all Fig4 variants tested confer rapamycin tolerance relative to no Fig4. Our data support a model wherein Fig4 that is not tethered to Vac14 engages in abnormal or sustained binding interactions with an unknown partner, thereby interfering with the normal cellular response to TORC1 inhibition by rapamycin. Whether these interactions reflect unknown mechanisms of Fig4 influence on the regulation of PI3,5P2 or direct physical interaction of Fig4 with pathways unrelated to PI3,5P2 production remains to be determined.

## 2 Results

### 2.1 Multiple Disease‐Related Variants of Fig4 Confer Tolerance to Rapamycin

Growth in *S. cerevisiae* is not limited by Fig4 function under normal growth conditions (Giaever et al. 2002); haploid Fig4 knockout yeast cells expressing wild‐type Fig4 or disease‐related Fig4 variants from a plasmid grow identically to cells expressing no Fig4 (Duex, Nau, et al. 2006; Strunk et al. 2020). In a survey for stress conditions under which Fig4 might be important for growth, we were surprised to find that, in the presence of 10 nM rapamycin at 37°C, Fig4 mutants associated with multiple human diseases enhanced growth relative to wild‐type (Figure 1B). Fig4 point mutants conferring rapamycin tolerance included Fig4‐I59T (CMT4J, ALS, and PLS), Fig4‐E72Y (ALS), and Fig4‐Y181S (leukodystrophy). A C‐terminal truncation, Fig4‐ΔCt113, which corresponds to a human Fig4 variant associated with leukodystrophy that is missing the last 115 amino acids as the result of a splice junction mutation, also conferred rapamycin tolerance (Lenk et al. 2019) (Figure 1A). This is not a comprehensive list of Fig4 disease‐related mutations; therefore, this phenotype is not generalizable to all Fig4 disease‐related variants. The strength of the phenotype varied between mutants, with the N‐terminal mutations, Fig4‐I59T and Fig4‐E72Y, displaying the most prominent growth advantage. Rapamycin tolerance conferred by Fig4 mutants was also observed at the slightly less stringent growth temperature of 36°C. At this temperature, cells expressing wild‐type or no Fig4 are not fully growth‐arrested; they continue to divide at a slower rate, suggesting that the growth advantage conferred by disease‐related mutants persists over prolonged rapamycin exposure (Figure 1C). Importantly, cells expressing no Fig4 displayed a growth rate similar to wild‐type under these conditions, indicating that rapamycin tolerance is not a result of loss of Fig4 function but rather involves the presence of these Fig4 mutants engaging in behavior different from wild‐type. Although the relative growth pattern remained the same between Fig4 variants, growth varied slightly from experiment to experiment, potentially due to minor variations in temperature and humidity.

### 2.2 Rapamycin Tolerance Conferred by Fig4 Mutants Is Temperature‐Dependent

To determine if Fig4 disease‐related mutants confer rapamycin tolerance across a wider range of temperatures, we repeated these assays at 30°C, 33°C, 36°C, and 37°C, ranging from standard growth temperature (30°C) to moderate heat stress (37°C). In these assays, we also included two additional mutants. The first is Fig4‐AAA, a rationally constructed mutant substituting three universally conserved threonines, T52, T62, and T78, with alanine on the N‐terminal surface near E72 and I59 (Figure 1A). Fig4‐AAA results in severely impaired binding of Fig4 to the Fab1‐Vac14‐Fig4 complex and failure to support PI3,5P2 elevation following hyperosmotic shock (Strunk et al. 2020). Also included was Fig4‐C467S, which is catalytically inactive as a result of substitution of the catalytic cysteine for serine (Rohde et al. 2003; Lees et al. 2020). At 30°C on 10 nM rapamycin, cells expressing disease‐related mutants do not display a growth advantage relative to no Fig4. Notably, at this temperature cells without Fig4 and cells expressing Fig4‐AAA display a relative growth advantage (Figure 1D). Similar to cells lacking Fig4, cells expressing Fig4‐AAA are expected to have little or no Fig4 associated with the Fab1‐Vac14‐Fig4 complex. This suggests that, in contrast to what is observed at 37°C, rapamycin tolerance at 30°C may result from a loss of Fig4 function rather than altered behavior of Fig4. As temperatures increase from 33°C to 37°C, the relative rapamycin tolerance observed in the absence of Fig4 is progressively diminished. In contrast, the relative tolerance conferred by Fig4 disease‐related variants emerges as the temperature is raised above 33°C (Figure 1D). Remarkably, cells expressing Fig4‐C467S displayed enhanced rapamycin sensitivity compared with Fig4‐WT across all temperatures (Figure 1D).

### 2.3 Fig4 Mutants That Confer Rapamycin Tolerance Display Decreased Association With the Fab1‐Vac14‐Fig4 Complex

**FIGURE 2:**
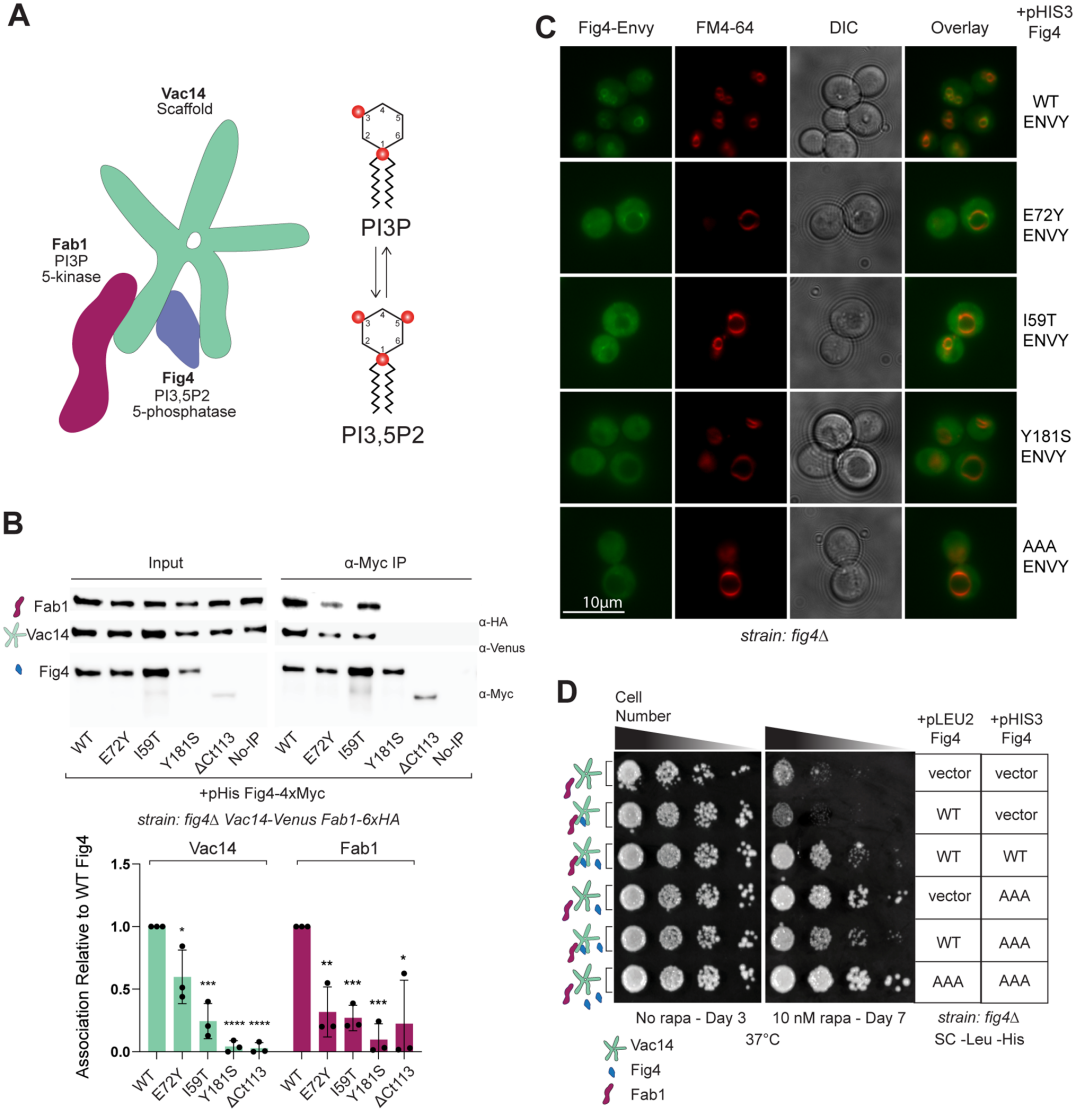
Rapamycin tolerance conferred by Fig4 disease‐related mutants is associated with impaired binding to the Fab1‐Vac14‐Fig4 complex and is dominant to expression of Fig4‐WT. (A) Cartoon representation of Fab1‐Vac14‐Fig4 complex adapted from Lees et al. (2020). Fig4 (~100 kDa) and Fab1 (~260 kDa) monomers associate with Vac14 pentamer (~100 kDa ×5) to support the production and turnover of PI3,5P2 (Fig4 phosphatase activity removes the inositol 5‐phosphate of PI3,5P2 to produce PI3P, Fab1 kinase activity performs the reverse reaction). (B) Fig4 disease‐related variants display impaired association with Fab1 and Fig4. Western blot of proteins immunoprecipitated with anti‐Myc antibody (IP) from a *fig4Δ Vac14‐Venus Fab1‐6xHA* strain transformed with plasmids expressing the indicated 4xMyc‐tagged Fig4 variants or untagged Fig4 wild‐type (WT‐tagless) as a negative control. *Vac14‐Venus* and *Fab1‐6xHA* are expressed from endogenous loci. Vac14 and Fab1 associated with Fig4 variants quantified relative to Fig4‐WT and normalized to Fig4 in each IP, with data points and mean (bar) from three independent experiments. Error bars represent standard deviation (ns, not significant; *p < 0.05, **p < 0.01, ***p < 0.001, ****p < 0.0001 by two‐tailed *t*‐test). (C) Fig4 disease‐related variants display diffuse cytoplasmic localization relative to Fig4‐WT. Fluorescence micrographs of a *fig4Δ* strain transformed with plasmids expressing indicated C‐terminally Envy‐tagged Fig4 variants. Vacuoles were stained with FM4‐64. (D) Rapamycin tolerance conferred by Fig4 variants is dominant to wild‐type Fig4 and is also conferred by wild‐type Fig4 when in excess. A *fig4Δ* strain transformed with the indicated plasmids was spotted on agar plates and grown under the conditions specified. Fig4 variants in this figure: Wild‐type (WT), CMT4J (I59T), ALS (E72Y), and leukodystrophy (Y181S and ΔCt113), T52A‐T62A‐T78A (AAA), catalytically dead (C467S), or no Fig4 (vector). All genes expressed from plasmids with native 5′ and 3′ regulatory regions.

Known Fig4 functions, including facilitation of both the production and turnover of PI3,5P2, involve its physical association with Fab1 and Vac14 (Lees et al. 2020; Rivero‐Ríos and Weisman 2022) (Figure 2A). Previous work indicated that Fig4‐I59T and Fig4‐E72Y are impaired in binding to the Fab1‐Vac14‐Fig4 complex and do not support full elevation of PI3,5P2 following hyperosmotic shock (Chow et al. 2009; Lenk et al. 2011). We hypothesized that the impaired ability to support the assembly of the Fab1‐Vac14‐Fig4 complex might be a shared property of cells expressing the Fig4 mutants that confer rapamycin tolerance. To investigate this possibility, we performed immunoprecipitations of Fab1 and Vac14 from cell lysates with these Fig4 variants. Each of the disease‐related Fig4 mutants displaying rapamycin tolerance: I59T, E72Y, Y181S, and ΔCt113 displayed reduced association with Fab1 and Vac14 relative to wild‐type in this assay (Figure 2B). To assess whether disease‐related variants of Fig4 are mislocalized as a result of their decreased association with the Fab1‐Vac14‐Fig4 complex, we performed fluorescence microscopy of live cells expressing Envy‐tagged Fig4 variants (Slubowski et al. 2015). Fig4 mutants conferring rapamycin tolerance displayed weaker localization to the vacuole and more diffuse cytoplasmic distribution relative to Fig4‐WT, consistent with their impaired binding to Vac14 (Figure 2C). Cells expressing disease‐related Fig4 variants displayed slightly enlarged vacuoles (Figure 2C), an indication of reduced PI3,5P2 levels in these cells (Rudge et al. 2004; Duex, Tang, and Weisman 2006; Chow et al. 2007, 2009). Notably, the relative impairment of each Fig4 variant in binding to the Fab1‐Vac14‐Fig4 complex did not correlate directly with the degree of rapamycin tolerance. This suggests that, if weaker binding to or increased dissociation from Fab1 and Vac14 contributes to the ability of Fig4 variants to confer rapamycin tolerance, it is not the only factor.

### 2.4 Rapamycin Tolerance Conferred by Fig4 Mutants Is Dominant and Is Similarly Conferred by Excess Wild‐Type Fig4

The finding that impaired association with the Fab1‐Vac14‐Fig4 complex was a common characteristic of Fig4 mutants conferring rapamycin tolerance led us to hypothesize that tolerance was being conferred by unbound Fig4 engaging in interactions outside of the Fab1‐Vac14‐Fig4 complex. If this were true, it would be expected that Fig4‐AAA would be dominant to Fig4‐WT because Fig4 that is not associated with the Fab1‐Vac14‐Fig4 complex would be able to engage in these interactions, whether or not wild‐type Fig4 is present. To test this, we simultaneously expressed Fig4‐WT and Fig4‐AAA from separate centromeric plasmids. Supporting our model, Fig4‐AAA conferred rapamycin tolerance even in the presence of Fig4‐WT, albeit to a lesser degree than Fig4‐AAA alone (Figure 2D). We further speculated that if the presence of Fig4 outside of the Fab1‐Vac14‐Fig4 complex was sufficient to confer rapamycin tolerance, Fig4‐WT should similarly be able to confer this phenotype if there were enough copies present to saturate the Fig4 binding capacity of the Fab1‐Vac14‐Fig4 complex. Indeed, modestly increasing the cellular level of Fig4 protein by expressing two copies of Fig4‐WT from two centromeric plasmids similarly interfered with rapamycin‐induced growth inhibition (Figure 2D, Figure S1). Moreover, two copies of Fig4‐AAA enhanced growth beyond Fig4‐AAA alone, suggesting that rapamycin tolerance can be further enhanced by increasing concentrations of Fig4 outside of the Fab1‐Vac14‐Fig4 complex (Figure 2D, Figure S1), consistent with Fig4 conferring tolerance by binding to something outside of the complex.

### 2.5 Fig4‐Dependent Tolerance to Rapamycin Does Not Require Fig4 Catalytic Function

**FIGURE 3:**
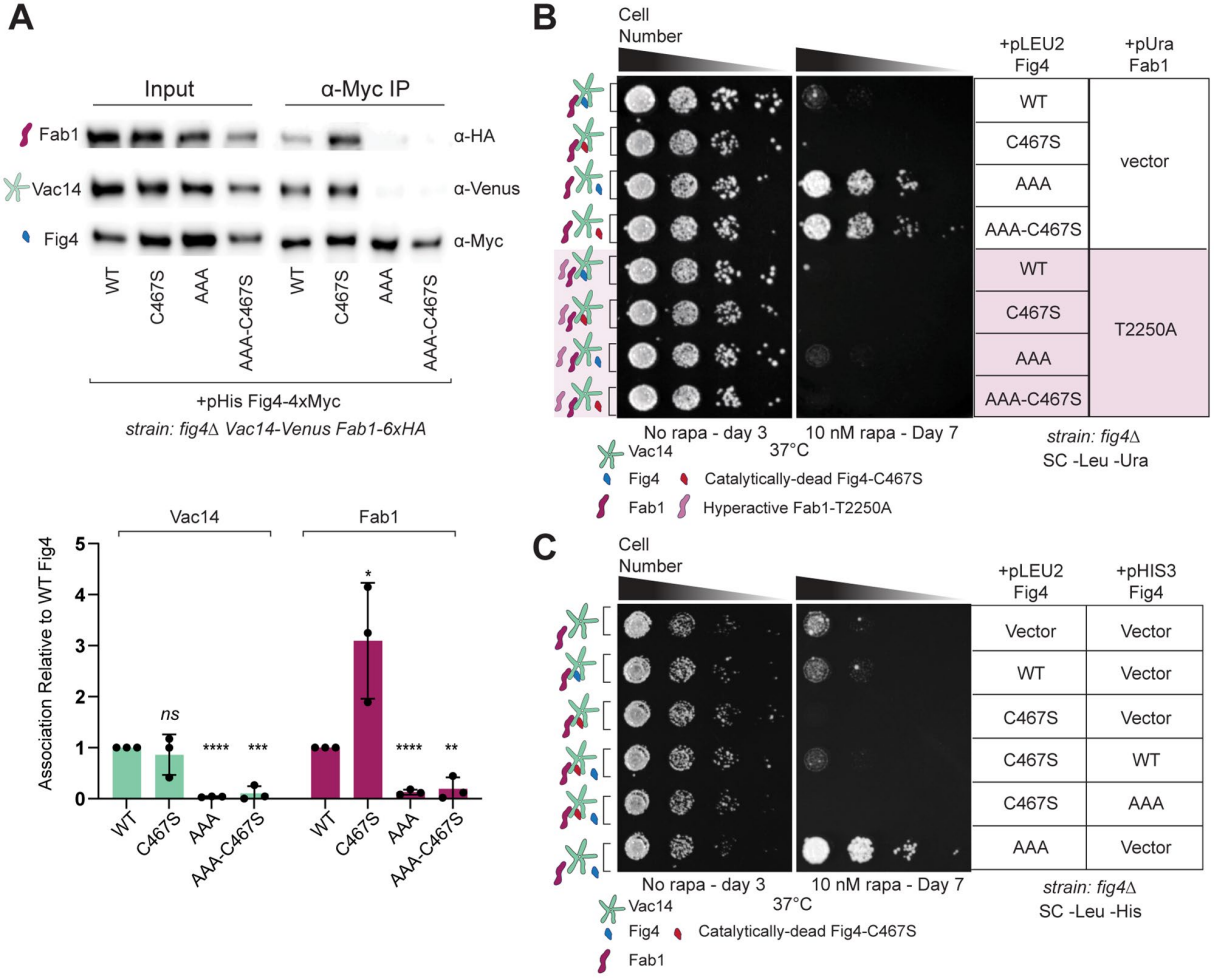
Fig4 catalytic function is not required for rapamycin tolerance at 37°C. (A) Catalytically dead Fig4‐C467S‐T52A‐T62A‐T78A (AAA‐C467S) is impaired in binding to Fab1 and Vac14. Western blot of proteins immunoprecipitated (IP) with anti‐Myc antibody from a *fig4Δ Vac14‐Venus Fab1‐6xHA* strain transformed with plasmids expressing indicated 4xMyc‐tagged Fig4 variants or untagged Fig4 wild type (WT‐tagless) Vac14‐Venus and Fab1‐6xHA are expressed from endogenous loci. Vac14 and Fab1 associated with Fig4 variants quantified relative to Fig4‐WT and normalized to Fig4‐4xMyc in each IP, with data points and mean (bar) from three independent experiments. Error bars represent standard deviation (ns, not significant; *p < 0.05, **p < 0.01, ***p < 0.001, ****p < 0.0001 by two‐tailed *t*‐test). (B) Catalytically dead Fig4 impaired in binding to Fab1 and Vac14 confers tolerance to rapamycin. A *fig4Δ* strain transformed with indicated plasmids was spotted on agar plates and grown under the conditions indicated. (C) Rapamycin sensitivity conferred by catalytically dead Fig4‐C467S is rescued by wild type Fig4 but not a mutant impaired in association with the Fab1‐Vac14‐Fig4 complex. Growth assays performed as in (B). Fig4 variants in this figure: Wild type (WT), catalytically dead (C467S), T52A‐T62A‐T78A (AAA), catalytically dead C467S‐T52A‐T62A‐T78A (AAA‐C467S), or no Fig4 (vector). All genes expressed from plasmids with native 5′ and 3′ regulatory regions.

**FIGURE 4:**
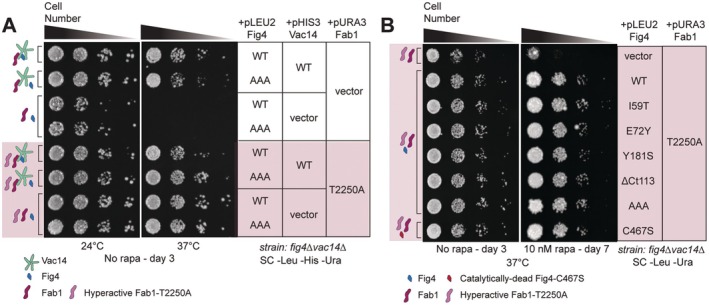
Fig4 variants confer rapamycin tolerance independent of Vac14. (A) Temperature sensitivity of a *fig4Δvac14Δ* strain is rescued by expression of hyperactive Fab1‐T2250A. A *fig4Δvac14Δ* strain was co‐transformed with plasmids expressing the indicated Fig4 variants or no Fig4 (vector), wild‐type Vac14 or no Vac14, hyperactive Fab1 (T2250A—pink shading), or no Fab1. Cells were spotted on agar plates in a 10‐fold dilution series and grown under the conditions specified. (B) Expression of any Fig4 variant confers tolerance to rapamycin at 37°C. Growth assay performed as in (A). Fig4 variants in this figure: Wild‐type (WT), T52A‐T62A‐T78A (AAA), catalytically‐dead (C467S), or no Fig4 (vector). All genes expressed from plasmids with native 5′ and 3′ regulatory regions.

The observation that expression of catalytically dead Fig4‐C467S resulted in enhanced sensitivity to rapamycin at all temperatures tested (see Figure 1D) raised the question of whether Fig4 catalytic function is required for the ability of Fig4 mutants to confer rapamycin tolerance under these conditions. The catalytically dead Fig4 mutant Fig4‐C467S is not impaired in association with the Fab1‐Vac14‐Fig4 complex; in fact, this mutant displays enhanced association with Fab1 (Strunk et al. 2020) (Figure 3A). Given the correlation between defects in association of Fig4 mutants with the Fab1‐Vac14‐Fig4 complex and their ability to confer rapamycin tolerance, we sought to generate a catalytically dead Fig4 mutant simultaneously impaired in binding to the Fab1‐Vac14‐Fig4 complex. We introduced the serine substitution of the Fig4 catalytic cysteine into the Fig4‐AAA variant to make Fig4‐AAA‐C467S. Fig4‐AAA‐C467S was impaired in binding to the Fab1‐Vac14‐Fig4 complex to a similar degree as Fig4‐AAA (Figure 3A) and conferred rapamycin tolerance on par with that conferred by Fig4‐AAA (Figure 3B). These data suggest that rapamycin tolerance conferred by Fig4 mutants is independent of its ability to turn over PI3,5P2 or any other potential catalytic function.

### 2.6 Variants of Fab1 and Fig4 Associated With Increased PI3,5P2 Production Display Rapamycin Sensitivity

In contrast to cells that lack Fig4, which are deficient in the elevation of PI3,5P2 following hyperosmotic stress (Duex, Nau, et al. 2006), cells expressing Fig4‐C467S have elevated basal levels of PI3,5P2 compared to wild‐type and display sustained PI3,5P2 elevation following stress (Strunk et al. 2020). This is because Fig4‐C467S confers the ability to support PI3,5P2 elevation, but lacks the catalytic activity to turn it over. We speculated that rapamycin sensitivity displayed by cells expressing Fig4‐C467S could be a result of chronic elevation of PI3,5P2. We therefore tested how the elevation of PI3,5P2 through an alternative mechanism, the expression of the hyperactive mutant Fab1‐T2250A, influenced growth on rapamycin. Fab1‐T2250A was shown previously to display elevated basal levels of PI3,5P2 and to bypass the requirement for Fig4 in the elevation of PI3,5P2 following hyperosmotic shock (Duex, Tang, and Weisman 2006; Lang et al. 2017). Cells expressing Fab1‐T2250A showed increased sensitivity to rapamycin regardless of the Fig4 mutant being expressed at 37°C (Figure 3B) as well as lower temperatures (Figure S2). Notably, simultaneous expression of Fig4‐AAA in cells expressing Fig4‐C467S did not rescue rapamycin sensitivity (Figure 3C). This finding ruled out the possibility that reduced availability of free Fig4 in cells expressing Fig4‐C467S caused rapamycin sensitivity. In contrast, restoring Fig4 catalytic function to cells expressing Fig4‐C467S by co‐expressing Fig4‐WT rescued rapamycin sensitivity (Figure 3C). This supports the likelihood that the failure of Fig4 engaged in the Fab1‐Vac14‐Fig4 complex to dephosphorylate a substrate is responsible for the rapamycin sensitivity imposed by Fig4‐C467S. Taken together, our data are consistent with the possibility that sustained elevation of PI3,5P2 leads to rapamycin sensitivity.

### 2.7 Fig4 Confers Rapamycin Tolerance in the Absence of Vac14

Available data suggest that the association between Fig4 and Fab1 requires direct binding between Fig4 and the scaffold protein Vac14 (Jin et al. 2008; Lees et al. 2020). If rapamycin tolerance is conferred by Fig4 not engaged with the Fab1‐Vac14‐Fig4 complex, any Fig4 variant would be expected to confer rapamycin tolerance in the absence of Vac14. Testing this prediction is complicated by the fact that cells lacking Vac14 do not grow at 37°C due to PI3,5P2 insufficiency (Gomes de Mesquita et al. 1996; Bonangelino et al. 1997; Murén et al. 2001; Dove et al. 2002). To rescue growth in a *fig4Δvac14Δ* strain at 37°C, we expressed the hyperactive Fab1‐T2250A mutant to restore sufficient PI3,5P2 production in these cells (Duex, Tang, and Weisman 2006) (Figure 4A). In this rescued background lacking Vac14, all Fig4 variants tested, including Fig4‐WT and Fig4‐C467S, were more tolerant to rapamycin at 37°C than cells expressing no Fig4 (Figure 4B). Although there is no reported evidence for Fig4 associating with Fab1 in the absence of Vac14, we cannot rule out the possibility that Fig4 confers rapamycin tolerance through an uncharacterized interaction with Fab1. Regardless, the data demonstrate that Fig4 enhances growth on rapamycin independent of Vac14 and its known mode of association with Fab1.

## 3 Discussion

**FIGURE 5:**
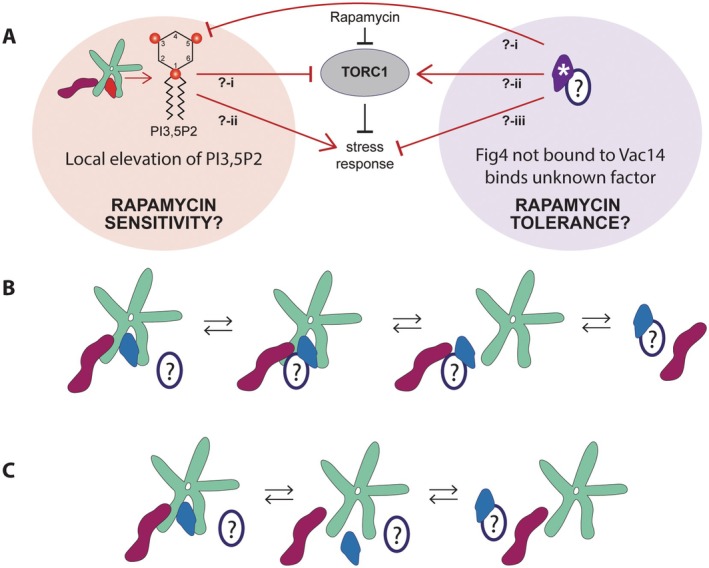
Hypothetical models for Fig4‐mediated rapamycin tolerance (A) Fig4 impaired in association with the Fab1‐Vac14‐Fig4 complex (purple with asterisk) may confer rapamycin tolerance by binding to an unknown factor (white oval with question mark). Fig4 binding to the unknown factor could mitigate the effect of rapamycin treatment by (i) altering PI3,5P2 production in a manner that enhances TORC1 signaling, (ii) reducing TORC1 inhibition, or (iii) altering the behavior of pathways that control growth downstream of TORC1 inhibition. Conversely, rapamycin sensitivity may be caused by chronic elevation of PI3,5P2 which could either (i) enhance TORC1 inhibition or (ii) alter the behavior of pathways that control growth downstream of TORC1 inhibition. Chronic elevation of PI3,5P2 may be induced by expression of hyperactive Fab1 or catalytically‐dead Fig4 (red), which supports PI3,5P2 production by the Fab1‐Vac14‐Fig4 complex without the ability to turn it over again. (B) Example hypothetical mechanism for Fig4‐dependent influence on PI3,5P2 production without binding Vac14. Fig4 that is not associated with the complex could stabilize or block Fab1 association with an unknown factor that they normally both directly contact within the context of the Fab1‐Vac14‐Fig4 complex. (C) Hypothetical mechanism for Fig4 influence on homeostasis through an interaction with an unknown factor that is mutually exclusive with its participation in the Fab1‐Vac14‐Fig4 complex.

Fig4 is believed to function in the production and turnover of PI3,5P2 through its participation in the Fab1‐Vac14‐Fig4 complex via direct interaction with Vac14. Here we report that, compared to wild‐type or no Fig4, multiple disease‐related mutants of Fig4, which are impaired in binding to the Fab1‐Vac14‐Fig4 complex, display rapamycin tolerance at high growth temperatures. Rapamycin tolerance is not a result of total loss of Fig4 function, as cells expressing no Fig4 grow at a rate similar to wild‐type. Moreover, all Fig4 variants tested confer rapamycin tolerance compared to no Fig4 in the complete absence of Vac14. These data suggest that, at least in this context, Fig4 is interacting with something other than Vac14 to influence cellular homeostasis. Importantly, rapamycin tolerance is conferred independent of Fig4 catalytic function. We propose that mutations that result in increased Fig4 outside of the Fab1‐Vac14‐Fig4 complex alter TORC1 signaling in yeast through context‐inappropriate or abnormally sustained binding of free Fig4 with an unknown factor. This change in TORC1 signaling could be mediated either via altered PI3,5P2 production, or through unrecognized Fig4 interactions that influence cellular homeostasis independent of changes in PI3,5P2 (Figure 5A).

It is unlikely that the Fig4 disease‐related mutants studied here directly interfere with the uptake or transport of rapamycin. If this were the case, their expression would be expected to prevent the rapamycin‐mediated inhibition of cell growth imposed by expression of the catalytically‐dead mutant Fig4‐C467S. Contrary to this, we found that rapamycin sensitivity persists when a Fig4 mutant that confers rapamycin tolerance on its own is simultaneously expressed with Fig4‐C467S (see Figure 3C). One possibility is that Fig4 mutants are interfering with signaling downstream of TORC1 inhibition, altering the response to loss of TORC1 activity (Figure 5A). For instance, Fig4 could interfere with entry into G1 arrest by blocking the function of the Cdk inhibitor Sic1 (Zinzalla et al. 2007), alter the regulation of amino acid transport (Jin et al. 2014; Neklesa and Davis 2008), or block stress‐related transcription programs that are normally upregulated when TORC1 is inhibited (Beck and Hall 1999). An alternative possibility is that Fig4 is acting upstream of TORC1, interfering with the full extent of TORC1 inhibition that is normally imposed by rapamycin treatment under these conditions (Neklesa and Davis 2008; Chen et al. 2020) (Figure 5A). Rapamycin is a noncompetitive inhibitor of TORC1, which acts through binding to the receptor protein, FKB1 (FKBP12 in humans). Binding of the FKB1‐rapamycin complex to TORC1 allosterically inhibits the catalytic subunit, Tor1/2 (Saxton and Sabatini 2017; Panwar et al. 2023). This leaves room for modulation of TORC1 signaling even when FKBP binding to rapamycin is saturated (Urban et al. 2007; Neklesa and Davis 2008).

PI3,5P2 is known to modulate TORC1 activity, although the mechanisms of its influence are not fully understood. TORC1 functions at endolysosomal membranes. In mammals, TORC1 is dynamically recruited to the endolysosomal membrane via binding of one of its subunits, Raptor, to PI3,5P2 (Bridges et al. 2012). In *S. cerevisiae*, the homolog of Raptor, Kog1, also binds PI3,5P2, but TORC1 is not dependent on PI3,5P2 for association with the vacuole. Nonetheless, PI3,5P2 production is critical for TORC1 function in *S. cerevisiae* as at least one key substrate, Sch9, is recruited to TORC1 on the vacuole via PI3,5P2 binding, and PI3,5P2 supports TORC1‐dependent regulation of autophagy and amino acid transport (Jin et al. 2014). PI3,5P2 may also modulate TORC1 function indirectly through activation of the vacuolar ATPase (Dechant et al. 2014; Rivero‐Ríos and Weisman 2022). Moreover, PI3P is consumed in the production of PI3,5P2; therefore, it is also possible that production of PI3,5P2 at high rates leads to displacement or altered function of TORC1 regulators and effectors that bind PI3P. For example, the critical TORC1 regulator PI3P‐binding protein 2 (Pib2) is modulated via PI3P binding (Hatakeyama 2021). Given the relationship between PI3,5P2 production and TORC1 function, changes in regulation of PI3,5P2 as a result of Fig4 mutation could reasonably explain the altered rapamycin sensitivity we observe. A temperature‐dependent mechanism of Fab1 regulation that can be influenced by Fig4 outside of the Fab1‐Vac14‐Fig4 complex would explain why we only observe Fig4‐dependent rapamycin tolerance at high growth temperatures. Similarly, the rapamycin sensitivity at all temperatures imposed by catalytically dead Fig4 and hyperactive Fab1 may be a consequence of their temperature‐independent elevation of PI3,5P2.

Yeast mutants shown previously to have opposing effects on PI3,5P2 production confer opposite phenotypes with regard to rapamycin sensitivity. Mutants that were previously associated with impaired elevation of PI3,5P2 (Chow et al. 2009; Lenk et al. 2011; Strunk et al. 2020) confer increased rapamycin tolerance. In contrast, mutants shown previously to chronically elevate global PI3,5P2 levels including catalytically‐dead Fig4 and hyperactive Fab1‐T2250A (Duex, Tang, and Weisman 2006; Lang et al. 2017; Strunk et al. 2020) result in increased rapamycin sensitivity. If elevation of PI3,5P2 can lead to rapamycin sensitivity, Fig4 disease‐related mutants could confer rapamycin tolerance by blocking increased or local activation of Fab1. This is a counterintuitive model as PI3,5P2 is required for yeast growth at high temperatures (Gary et al. 1998; Jin et al. 2017). Moreover, this model implies that moderate PI3,5P2 elevation contributes to growth inhibition under heat stress in the presence of rapamycin, even in wild‐type cells. Alternatively, Fig4 dissociated from Vac14 could support enhanced production of a specific, growth‐promoting pool of PI3,5P2 on the vacuole. In either case, independent of Vac14 or phosphatase activity, Fig4 would have to be engaging in a yet unrecognized interaction to influence Fab1 activity. This could involve blocking or stabilizing Fab1 binding to a regulatory factor on the vacuole that Fig4 normally contacts from within the Fab1‐Vac14‐Fig4 complex, perhaps Vac7, Atg18, or Fab1 itself (Botelho et al. 2008; Jin et al. 2008) (Figure 5B). Alternatively, Fig4 could confer rapamycin tolerance through an interaction that is mutually exclusive with its participation in the Fab1‐Vac14‐Fig4 complex (Figure 5C).

Interestingly, rapamycin sensitivity at 37°C is also divergently affected by different substitution mutations in amino acids in Fab1 reported to be phosphorylated by TORC1 (Chen et al. 2020). Phosphomimetic aspartate substitutions at these sites result in rapamycin sensitivity, which is associated with the shift of Fab1 from the vacuole membrane to PI3,5P2 dense, vacuole‐adjacent puncta known as signaling endosomes. Conversely, alanine substitutions at these phosphosites, mimicking the unphosphorylated state, result in rapamycin tolerance, which is associated with increased Fab1 dispersion on the larger vacuole membrane (Chen et al. 2020). Importantly, TORC1 colocalizes with Fab1 in both mutants. It is proposed that TORC1‐dependent phosphorylation of Fab1 results in its activation. High local Fab1 activity, in turn, may serve to alter TORC1 signaling, either through a decrease in PI3P or an increase in PI3,5P2 (Chen et al. 2020; Muneshige and Hatakeyama 2025; Nicastro et al. 2025). This would suggest that rapamycin tolerance conferred by Fig4 disease‐related mutants through interference with Fab1 activation, either by TORC1 or another activator, is a viable model for Fig4‐dependent rapamycin tolerance (Figure S3). An alternative possibility is that Fig4 outside of the Fab1‐Vac14‐Fig4 complex enhances growth‐promoting Fab1 activities favored when Fab1 is not phosphorylated at TORC1‐dependent sites.

It is also possible that Fig4 binding to a factor outside of the Fab1‐Vac14‐Fig4 complex could alter the response to rapamycin through a pathway that is independent of changes in PI3,5P2 production. Such a factor might only be expressed or impact growth at high temperatures, potentially only when TORC1 is inhibited. This model predicts that there are factors whose association with Fig4 is favored when Fig4 is not bound to Vac14 (Figure 5C). Whatever the mechanism, Fig4 could be disrupting normal regulation of TORC1 through a native binding interaction that is altered or sustained when Fig4 is not bound to Vac14. Determining the actual mechanism of Fig4‐dependent rapamycin tolerance might be facilitated by identifying proteins that can interact with Fig4 independent of Vac14, including known Fab1 regulatory factors, and testing whether they impact Fig4‐dependent rapamycin tolerance. Investigations of whether and how local regulation of PI3,5P2 production and TORC1‐dependent phosphorylation of substrates are influenced by Fig4 mutants will also be critical next steps.

The data reported here suggest that disease‐related mutants of Fig4 can interfere with the normal response to rapamycin in yeast through physiologically relevant binding interactions that are yet to be characterized. If such interactions are conserved in humans, there may be implications for understanding Fig4‐related disease pathology. Our findings suggest that, at least in *S. cerevisiae* , Fig4 disease‐related mutants can dominantly alter cellular homeostasis when co‐expressed with wild‐type Fig4 in haploid yeast (Figure 2D) suggesting a gain of function. In contrast to what we observe in yeast, Fig4 mutants impaired in binding to Vac14 are efficiently degraded in mammalian cells (Ikonomov et al. 2010; Lenk et al. 2011). Pathology is accordingly attributed to loss of function within the Fab1‐Vac14‐Fig4 (PIKfyve‐ArPIKfyve‐Sac3) complex. Nonetheless, Vac14‐independent roles for Fig4 have been considered previously (Ikonomov et al. 2013; Morioka et al. 2017; Qi et al. 2022). Single point mutations in Fig4 have also been reported to be associated with neurodegenerative diseases in an autosomal dominant manner in humans (Chow et al. 2009; Yilihamu et al. 2022). Given that individuals with a single functional copy of Fig4 are healthy (Nicholson et al. 2011; Campeau et al. 2013; Boura et al. 2024), it is possible that disease states associated with some Fig4 point mutations are not exclusively due to loss of Fig4 function. Investigating the mechanisms by which Fig4 disease‐related variants confer rapamycin tolerance may provide new understanding of the molecular mechanisms by which Fig4 contributes to cellular homeostasis.

## 4 Experimental Procedures

### 4.1 Yeast Strains and Plasmids

Strains and plasmids used in this study are listed in Table 1. All strains come from the same haploid yeast background, LSWY3250 (derived from SEY6210). All plasmids are centromeric (Sikorski and Hieter 1989). Yeast were grown in yeast extract–peptone–dextrose (1% yeast extract, 2% peptone, 2% dextrose; YEPD) or synthetic complete (SC) minimal medium without selective amino acids at the indicated strains, then transformed with plasmids expressing variants of Fig4, Vac14, and/or Fab1 from their native promoters and 5′ and 3′ UTRs. Transformed cells were grown overnight in selective SC media to mid–log phase. Cultures were diluted to ∼1.5 × 10^6^ cells per mL; a 10‐fold serial dilution was plated on selective SC agar plates (1.5%) using a 48‐Pin Microplate Replicator (V&P Scientific Inc.). Plates were grown at 24°C or 37°C for 3 days without rapamycin or 7–10 days with 10 nM rapamycin.

**TABLE 1:** Strains and plasmids used in this study.

Strain/plasmid	Genotype/description	Source
*fig4Δ*	*MATα, leu2, 3‐112, ura3‐52, his3‐Δ200, trp1‐Δ901, lys2‐801, suc2‐Δ9, fig4Δ::TRP1*	LWY6474 (Duex, Tang, and Weisman 2006)
*fig4Δvac14Δ*	*MATα, leu2, 3‐112, ura3‐52, his‐Δ200, trp1‐Δ901, lys2‐801, suc2‐Δ9, fig4Δ::TRP1, vac14Δ::TRP1*	LWY6538 (Duex, Tang, and Weisman 2006)
*fig4ΔVac14‐Venus Fab1‐6xHA*	*MATa, leu2, 3‐112, ura3‐52, his‐Δ200, trp1‐Δ901, lys2‐801, suc2‐Δ9, fig4Δ::TRP1, VAC14‐Venus::KAN, FAB1‐6xHA::NAT*	LWY15782 (Strunk et al. 2020)
pRS413 empty vector	*Yeast centromeric plasmid; HIS3*	Sikorski and Hieter (1989)
pRS415 empty vector	*Yeast centromeric plasmid; LEU2*	Sikorski and Hieter (1989)
pRS416 empty vector	*Yeast centromeric plasmid; URA3*	Sikorski and Hieter (1989)
pRS415‐Fig4‐WT	*Yeast centromeric plasmid; LEU2*	Jin et al. (2008)^a^
pRS415‐Fig4‐E72Y	*Yeast centromeric plasmid; HIS3*	Chow et al. (2009)^a^
pRS415‐Fig4‐I59T	*Yeast centromeric plasmid; LEU2*	Chow et al. (2007)^a^
pRS415‐Fig4‐Y181S	*Yeast centromeric plasmid; LEU2*	This study^a^
pRS415‐Fig4‐CtΔ113‐4xMyc	*Yeast centromeric plasmid; LEU2*	This study^a^
pRS415‐Fig4‐T52AT62AT78A	*Yeast centromeric plasmid; LEU2*	Strunk et al. (2020)^a^
pRS413‐Fig4‐4xMyc	*Yeast centromeric plasmid; HIS3*	Jin et al. (2008)^a^
pRS413‐Fig4‐4xMyc‐E72Y	*Yeast centromeric plasmid; LEU2*	This study^a^
pRS413‐Fig4‐4xMyc‐I59T	*Yeast centromeric plasmid; LEU2*	This study^a^
pRS413‐Fig4‐4xMyc‐Y181S	*Yeast centromeric plasmid; LEU2*	This study^a^
pRS413‐Fig4‐4xMyc‐AAA	*Yeast centromeric plasmid; LEU2*	Strunk et al. (2020)^a^
pRS413‐Fig4‐4xMyc‐C467S	*Yeast centromeric plasmid; LEU2*	Strunk et al. (2020)^a^
pRS413‐Fig4‐4xMyc‐C467S‐AAA	*Yeast centromeric plasmid; LEU2*	This study^a^
pRS416‐Fab1‐T2250A	*Yeast centromeric plasmid; URA3*	Duex, Tang, and Weisman (2006)^b^
pRS413‐Vac14	*Yeast centromeric plasmid; HIS3*	This study^c^
pRS413‐Fig4‐Envy	*Yeast centromeric plasmid; HIS3*	Strunk et al. (2020)^a^
pRS413‐Fig4‐Envy‐I59T	*Yeast centromeric plasmid; HIS3*	This study^a^
pRS413‐Fig4‐Envy‐E72Y	*Yeast centromeric plasmid; HIS3*	This study^a^
pRS413‐Fig4‐Envy‐Y181S	*Yeast centromeric plasmid; HIS3*	This study^a^
pRS413‐Fig4‐Envy‐AAA	*Yeast centromeric plasmid; HIS3*	This study^a^

^a^ 1170 nucleotides included 5′ and 746 nucleotides included 3′ to Fig4 open reading frame.

^b^ 1472 nucleotides included 5′ and 1297 nucleotides included 3′ to Fab1 open reading frame.

^c^ 256 nucleotides included 5′ and 477 nucleotides included 3′ to Vac14 open reading frame.

### 4.2 Fluorescence Microscopy

Yeast were grown in selective SC medium to an OD600 of 0.5 at 24°C and labeled with FM4‐64 (Vida and Emr 1995). Fig4 was visualized with a C‐terminal Envy tag (Slubowski et al. 2015; Strunk et al. 2020). Cells were harvested at 1000 ***g*** for 3 min at 24°C and resuspended in 250 μL YEPD. Six microliter FM4‐64 (BIOTIUM) at 2 mg/mL in DMSO was added, and cells were incubated with shaking at 24°C for 30 min. FM4‐64‐labeled cells were washed twice with 250 μL SC media, then resuspended in 4 mL room temperature selective SC media. One milliliter cells were sedimented in microfuge tubes at 1000 ***g*** for 3 min and gently resuspended in 20–50 μL media. 2–3 μL of cells were applied to glass micro slides (Leica 3800220) imaged under cover glass (Leica 3800101) with a Nikon TE300 Inverted Phase Contrast DIC fluorescent microscope using a Nikon Plan Apo VC 100× Oil Immersion Microscope Objective (n.a. 1.4—Nikon MRD01901) with Cargille Laser Liquid 5610 (Cargille Labs 20130,20127‐RCF). Images were captured with a Photometrics CoolSNAP HQ Monochrome camera using NIS‐Elements software interface.

### 4.3 Co‐Immunoprecipitations From Yeast

The yeast strain fig4ΔVac14‐VenusFab1‐6xHA was transformed with plasmids expressing variants of 4xMyc‐tagged Fig4 or tagless Fig4 and additional plasmids as indicated. Log‐phase cells (25 OD600 U) grown in 50 mL liquid culture at 24°C were harvested and lysed with 1/2 volume 0.5 mm diameter zirconia/silica beads (Biospec; 11079105z) and 3 volumes (3 μL per mg of cells) of IP buffer (50 mM Tris, pH 7.5, 120 mM NaCl, 10 mM EDTA, 1 mM EGTA, 5 mM 2‐glycerophosphate [Sigma; G9422], 1× complete protease inhibitor cocktail EDTA‐free [Roche; 11836170001], and 1× Protease Inhibitor Cocktail for use with fungal and yeast extracts [Sigma; P8215], 3 mM benzamidine, 1 μg/mL leupeptin, 2 μg/mL aprotinin, and 6 μg/mL chymostatin, 1 ug/mL pepstatin). All subsequent steps were carried out at 4°C. Cells were disrupted for 8 × 1 min with a SoniBeast—Small Sample Cell Disruptor (Biospec; 42105) with 2 min intervals in an ice water bath. Debris was removed by centrifugation for 5 min at 500 ***g***. The supernatant was mixed with 5% octyl‐glucoside (Sigma; O8001) in lysis buffer for a final concentration of 0.5% octyl‐glucoside and incubated for 30 min. Octyl‐glucoside–solubilized lysate was cleared by spinning at 13,000 ***g*** for 10 min. Supernatants were incubated with 0.8 μL per 100 μL lysate mouse anti‐Myc antibody clone 9E10 (Sigma; 05‐419) for 1 h. One hundred microlitre of each lysate was applied to 15 μL Protein‐G Sepharose beads (Cytiva; 17061801) equilibrated in lysis buffer and incubated with rocking for 1 h. Beads were washed three times with 500 μL of IP buffer containing 0.5% octyl‐glucoside, 1 mM benzamidine, 5 mM 2‐glycerophosphate, and 1× Roche Complete inhibitor cocktail. Bound protein was eluted by heating immunoglobulin G beads with 35 μL of denaturing urea SDS‐loading dye (1% SDS, 8 M urea, 10 mM Tris, pH 6.8, 10 mM EDTA, 0.01% bromophenol blue, 1× Roche Complete inhibitor cocktail, 1 mM benzamidine, 1 μg/mL leupeptin, 2 μg/mL aprotinin, and 6 μg/mL chymostatin, 1 μg/mL pepstatin and 5% 2‐Mercaptoethanol) at 85°C for 10 min and spun for 1 min at 1000 ***g*** prior to SDS–PAGE and Western blot analysis.

### 4.4 Quantification of Relative Levels of Fig4 Protein in Cells

Yeast were grown in selective SC medium to an OD600 of 0.5 at 24°C. One milliliter cells were harvested at 1000 ***g*** for 3 min at room temperature. Cells were lysed with 100 μL denaturing urea SDS‐loading dye (1% SDS, 8 M urea, 10 mM Tris, pH 6.8, 10 mM EDTA, 0.01% bromophenol blue, 1× complete protease inhibitor cocktail EDTA‐free (Roche; 11836170001), 1 mM benzamidine, 1 μg/mL leupeptin, 2 μg/mL aprotinin, and 6 μg/mL chymostatin, 1 μg/mL pepstatin and 5% 2‐Mercaptoethanol). Fifty microliter of 0.5 mm diameter zirconia/silica beads (Biospec; 11079105z) were added to each sample before cells were disrupted with a SoniBeast—Small Sample Cell Disruptor (Biospec; 42105) in 0.5 mL tubes for 10 min at 4°C. Debris was removed by centrifugation for 5 min at 500 ***g***. Tubes were immediately heated at 95°C for 10 min and spun for 1 min at 1000 ***g*** prior to SDS–PAGE and Western blot analysis.

### 4.5 Western Blot Analysis

Each sample was run on a 4%–20% SDS‐PAGE gel (BioRad; 4561096) and transferred onto nitrocellulose membranes (Cytiva; 10600096). The following primary antibodies were used to detect affinity tagged constructs: Fig4‐4xMyc with rabbit anti‐Myc at 1:1000 (Cell Signaling; 2278S), Fab1‐6xHA with rabbit anti‐HA at 1:1000 dilution in 5% nonfat dry milk in TBST (Cell Signaling; 3724S), and Vac14‐Venus with mouse anti‐GFP 1:1000 dilution in 5% nonfat dry milk in TBST (Sigma; 11814460001). Detection through chemiluminescence was implemented with horseradish peroxidase conjugated secondary antibodies (Jackson Immunoresearch Laboratories; donkey anti‐mouse IgG—102650‐014 or goat anti‐rabbit IgG—102645‐188 at 1:10,000 dilution in 5% nonfat dry milk in TBST) and ECL Prime Western Blotting Detection Reagents (Amersham; RPN2232). Chemiluminescent blots were imaged with a Licor Odyssey Fc Imager using LICORbio Image Studio Software. For Co‐immunoprecipitations, Fab1 and Vac14 bands were normalized to Fig4 in the corresponding immunoprecipitation. Association is expressed relative to Fig4‐Myc wild‐type in each experiment. For relative quantification of Fig4‐4xMyc in lysates, bands were normalized to PGK1 detected with a mouse anti‐Pgk1 antibody at 1:10,000 dilution in 5% nonfat dry milk in TBST (Invitrogen; 459250). Fig4 levels are expressed relative to wild‐type Fig4 expressed from a single centromeric plasmid (1 copy wild‐type).

### 4.6 Statistical Analysis

All statistical analysis was done using GraphPad Prism. Statistical comparison between Fig4 variants and wild‐type (for immunoprecipitations) or one copy Fig4‐WT (for determination of relative levels of one or two copies of Fig4 variants) was conducted using the two‐sided Student's *t* test, as indicated in the figure legends. Differences were considered significant if the *p* value was < 0.05 (ns, not significant; *p < 0.05, **p < 0.01, ***p < 0.001, ****p < 0.0001).

## Author Contributions

**Hannah E. Reeves:** investigation, conceptualization. **Anna King:** investigation, formal analysis, writing – review and editing, visualization. **Imran Khan:** investigation, conceptualization. **Asha Thomas:** investigation. **Corey Chung:** investigation. **Anirudan Sivaprakash:** investigation, visualization, writing – review and editing. **Harrison A. Hall:** investigation. **Cole McGuire:** investigation, writing – review and editing. **Victoria Cruz:** investigation, writing – review and editing. **Alim Habib:** investigation. **Lauren Dotson:** visualization, investigation. **Sophia R. Lee:** investigation. **Caroline L. Darbro:** investigation. **Bethany S. Strunk:** conceptualization, investigation, writing – original draft, funding acquisition, supervision.

## Conflicts of Interest

The authors declare no conflicts of interest.

## Supporting information


**Figure S1:** mmi70018‐sup‐0001‐FiguresS1‐S3.pdf.
**Figure S2:** mmi70018‐sup‐0001‐FiguresS1‐S3.pdf.
**Figure S3:** mmi70018‐sup‐0001‐FiguresS1‐S3.pdf.

## Data Availability

The data that support the findings of this study are available from the corresponding author upon reasonable request.
