# Supplementary material for: Homeostatic Influence of Fig4 Outside of the Fab1‐Vac14‐Fig4 Complex in Saccharomyces cerevisiae
Source: Mol Microbiol. 2025 Jul 31;124(5):386–99. doi: 10.1111/mmi.70018 (PMC12594618; doi:10.1111/mmi.70018)
Supplement: Supplementary file 1 — Figure S1: mmi70018‐sup‐0001‐FiguresS1‐S3.pdf. Figure S2: mmi70018‐sup‐0001‐FiguresS1‐S3.pdf. Figure S3: mmi70018‐sup‐0001‐FiguresS1‐S3.pdf. [file MMI-124-386-s001.pdf]

Homeostatic influence of Fig4 outside of the Fab1-Vac14-Fig4 complex in *Saccharomyces cerevisiae*

Hannah E. Reeves[1]†, Anna King[1]†, Imran Khan[1]†, Asha Thomas[1], Corey Chung[1], Anirudan Sivaprakash[1], Harrison A. Hall[1], Cole McGuire[1], Victoria Cruz[1], Alim Habib[2], Lauren Dotson[1], Sophia R. Lee[1], Caroline L. Darbro[1], Bethany S. Strunk[1]

[1]Department of Biology, Trinity University, San Antonio, TX 78212
[2]Life Sciences Institute, University of Michigan, Ann Arbor, MI 48109
†These authors contributed equally to this study

Supplementary Information:

Figure S1

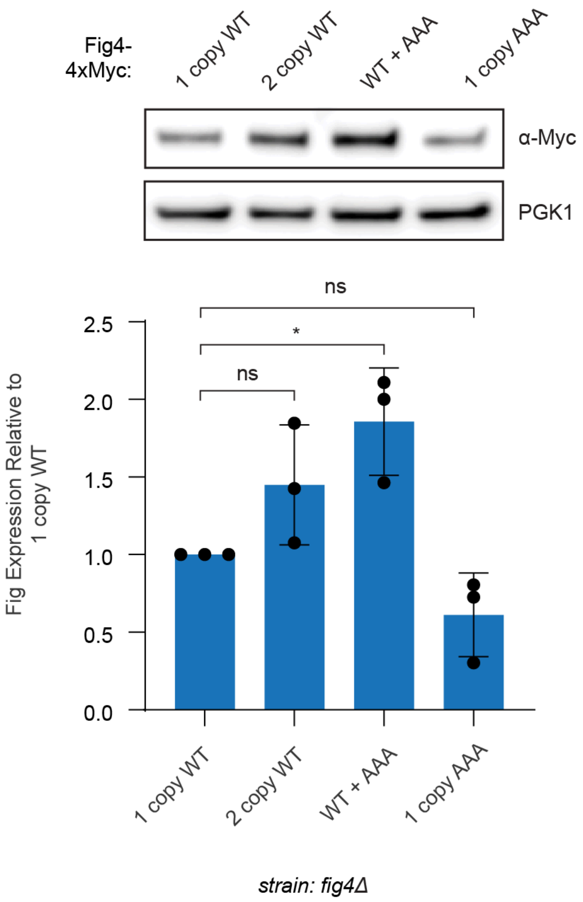

Figure S1: Relative levels of Fig4 in cells expressing one or two copies of Fig4. Western blot of proteins from denaturing lysis of a *fig4Δ* strain co-transformed with centromeric plasmids pRS415 (+pLEU2) and pRS413 (+pHIS3) expressing the indicated Fig4 4x-Myc-tagged variants or no Fig4 (vector). Plasmid-based Fig4 was expressed with native promoters and 5′ and 3′ UTRs. Bar graph shows quantification of Fig4 band intensities in lysates relative to 1 copy of Fig4 wild-type. Error bars represent standard deviation (*ns* not significant, $*p < 0.05$ by two-tailed t-test).

Figure S2

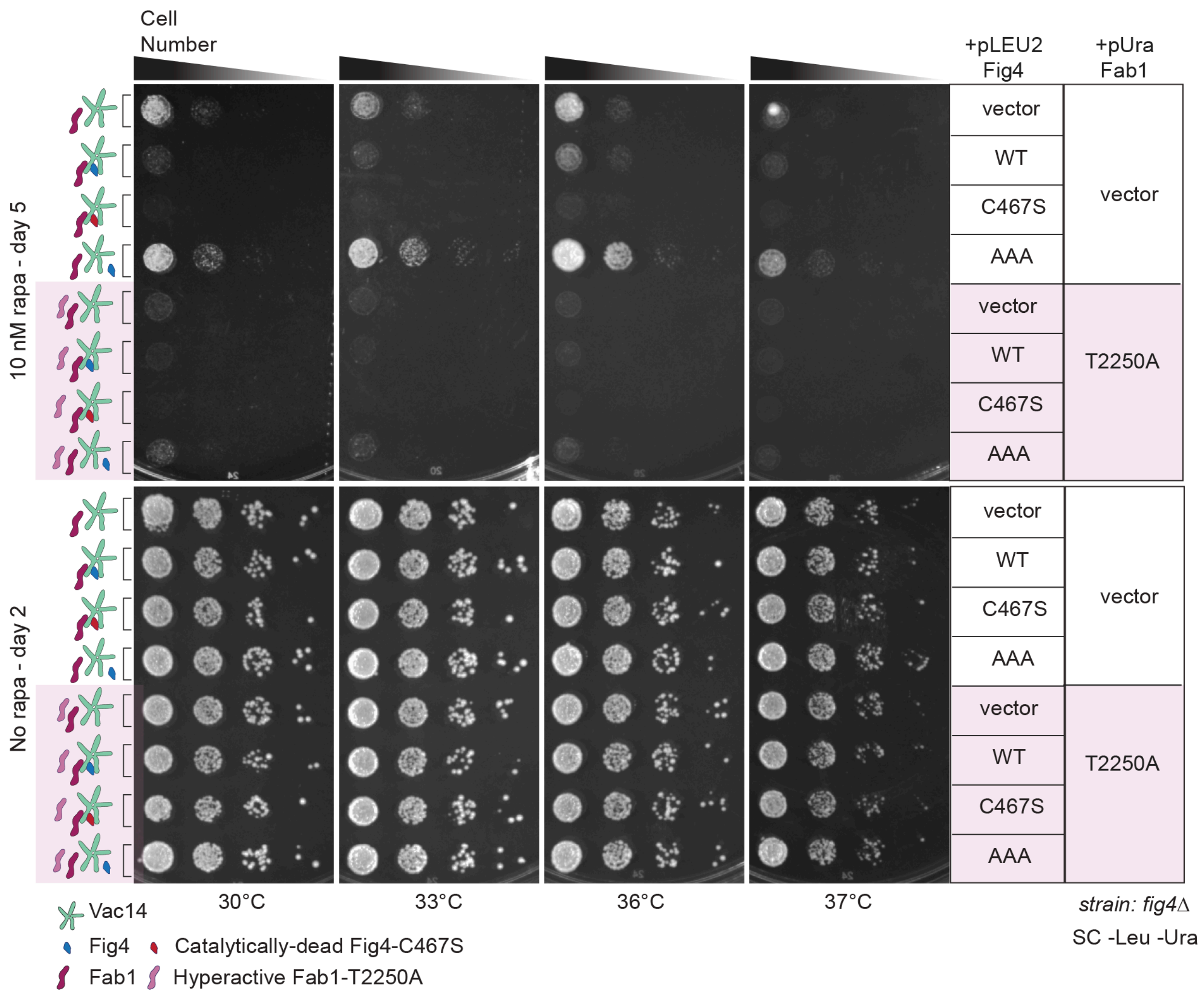

Figure S2: Expression of hyperactive Fab1 rapamycin sensitivity at standard and high growth temperatures. A *fig4Δ* strain was co-transformed plasmids expressing the indicated Fig4 variants, or no Fig4 (vector), and hyperactive Fab1 (T2250A - pink shading), or no Fab1. Cells were spotted on agar plates in a 10-fold dilution series at the temperatures indicated. Fig4 variants in this figure: Wild-type (WT), T52A-T62A-T78A (AAA), catalytically-dead (C467S), or no Fig4 (vector). All genes expressed from plasmids with native 5' and 3' regulatory regions.

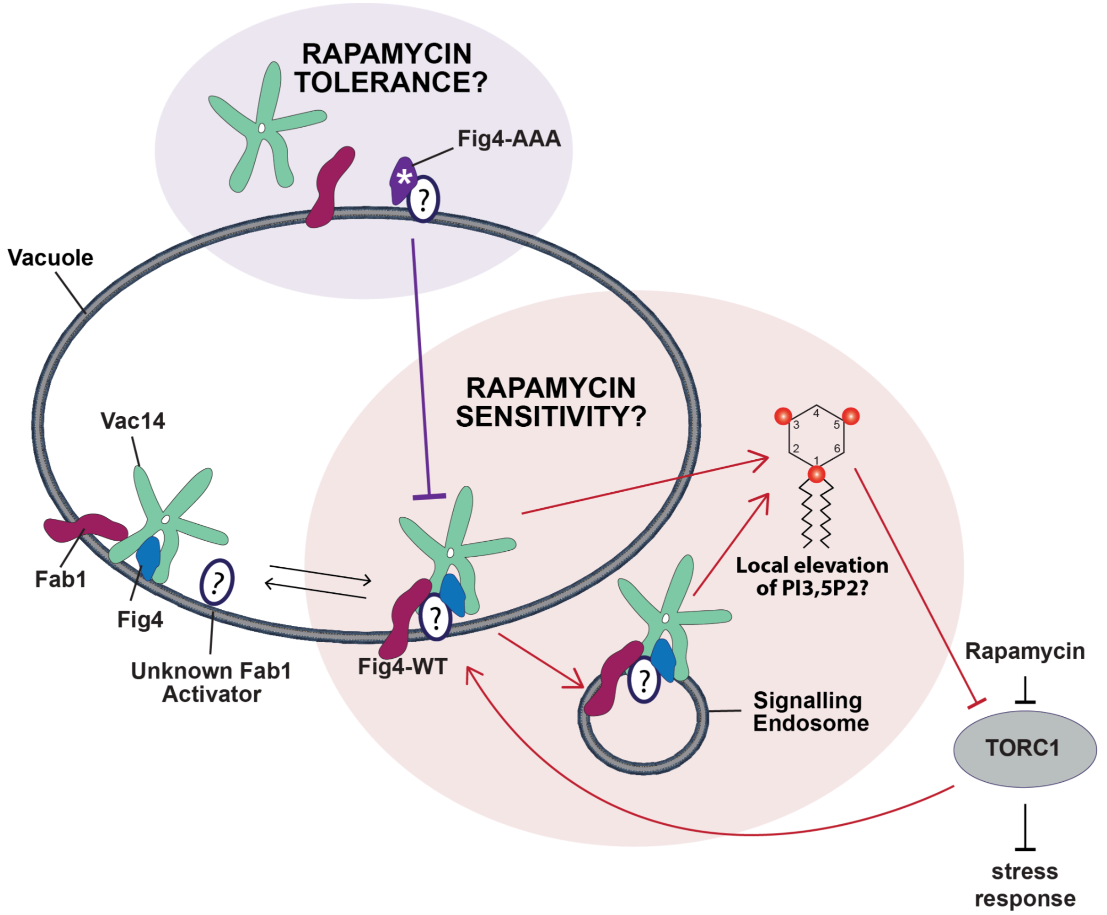

Figure S3: Hypothetical model for Fig4-mediated rapamycin tolerance through blocking Fab1 activation. This model requires two assumptions: 1) local elevation of PI3,5P2 in the vicinity of TORC1 restricts its ability to phosphorylate target substrates following rapamycin treatment, 2) Fab1 binding to an unknown Fab1 activating factor leads to local elevation of PI3,5P2. In this model, TORC1-dependent phosphorylation of Fab1 (Chen et al., 2020) or another mechanism of Fab1 activation (e.g. heat-stress dependent activation) stabilizes binding of the Fab1-Vac14-Fig4 complex to an unknown Fab1 activator. Binding to that Fab1 activator by the Fab1-Vac14-Fig4 complex involves direct contacts between both Fig4 and Fab1. Elevation of PI3,5P2 production as a result of Fab1 association with the unknown activator results in rapamycin sensitivity either by shifting Fab1 away from vacuolar targets (e.g. to signalling endosomes) or by altering TORC1 signalling via regulators or effectors that bind PI3,5P2 or PI3P. Fig4 mutants impaired in binding to the Fab1-Vac14-Fig4 complex (purple with asterisk) independently bind the unknown activator in a manner that interferes with Fab1 binding. Persistent blocking of Fab1 activation by Fig4 binding the unknown activator blocks PI3,5P2 elevation and thereby promotes rapamycin tolerance. Alternatively, Fig4 outside of the Fab1-Vac14-Fig4 complex could be enhancing production of a localized growth promoting pool of PI3,5P2 by Fab1.
